# Enteric *Aeromonas* Infection: a Common Enteric Bacterial Infection with a Novel Infection Pattern Detected in an Australian Population with Gastroenteritis

**DOI:** 10.1128/spectrum.00286-23

**Published:** 2023-06-28

**Authors:** Christopher Yuwono, Michael C. Wehrhahn, Fang Liu, Li Zhang

**Affiliations:** a School of Biotechnology and Biomolecular Sciences, University of New South Wales, Sydney, Australia; b Douglass Hanly Moir Pathology, Macquarie Park, Sydney, Australia; Quest Diagnostics

**Keywords:** *Aeromonas*, *Campylobacter*, *Salmonella*, *Shigella*, enteroinvasive *E. coli*, *Yersinia*, gastroenteritis

## Abstract

*Aeromonas* species are emerging human enteric pathogens. However, they are currently not routinely detected in many diagnostic laboratories, and information regarding *Aeromonas* enteric infections detected using molecular methods is lacking. Here, we investigated the detection of *Aeromonas* species and four other enteric bacterial pathogens in 341,330 fecal samples from patients with gastroenteritis processed in a large Australian diagnostic laboratory between 2015 and 2019. These enteric pathogens were detected using quantitative real-time PCR (qPCR) methods. Furthermore, we compared the qPCR cycle threshold (*C_T_*) values obtained from fecal samples that tested positive for *Aeromonas* only by molecular detection with those of samples that tested positive by both molecular detection and bacterial isolation methods. *Aeromonas* species were found to be the second most common bacterial enteric pathogens among patients with gastroenteritis. We observed a unique pattern of three infection peaks for *Aeromonas*, which correlated with the age of the patients. *Aeromonas* species were the most common enteric bacterial pathogens in children younger than 18 months. Fecal samples that tested positive for *Aeromonas* only by molecular detection had significantly higher *C_T_* values than fecal samples that tested positive by both molecular detection and bacterial culture. In conclusion, our findings reveal that *Aeromonas* enteric pathogens exhibit an age-related three-peak infection pattern, distinguishing them from other enteric bacterial pathogens. Moreover, the high rate of *Aeromonas* enteric infection discovered in this study suggests that *Aeromonas* species should be routinely tested in diagnostic laboratories. Our data also show that combining qPCR with bacterial culture can enhance the detection of enteric pathogens.

**IMPORTANCE** *Aeromonas* species are emerging human enteric pathogens. However, these species are currently not routinely detected in many diagnostic laboratories, and no studies have reported the detection of *Aeromonas* enteric infection using molecular methods. We investigated the presence of *Aeromonas* species and four other enteric bacterial pathogens in 341,330 fecal samples from patients with gastroenteritis using quantitative real-time PCR (qPCR) methods. Interestingly, we discovered that *Aeromonas* species were the second most common bacterial enteric pathogens in patients with gastroenteritis, exhibiting a novel infection pattern compared to those of other enteric pathogens. Furthermore, we found that *Aeromonas* species were the most prevalent enteric bacterial pathogens in children aged 6 to 18 months. Our data also revealed that qPCR methods exhibit higher sensitivity in detecting enteric pathogens compared to that of bacterial culture alone. Moreover, combining qPCR with bacterial culture enhances the detection of enteric pathogens. These findings emphasize the importance of *Aeromonas* species in public health.

## INTRODUCTION

*Aeromonas* species are Gram-negative, rod-shaped bacteria commonly found in aquatic environments (1–5). *Aeromonas* species are human pathogens that cause a variety of diseases, such as wound infections, bacteremia, and gastroenteritis (6–16). Gastrointestinal infection is the most prevalent form of disease caused by *Aeromonas* species in humans, ranging from mild self-limiting watery diarrhea to severe chronic infections that require antibiotic treatment (17–23). Approximately 40% of *Aeromonas*-induced gastrointestinal infections required antibiotic treatment in one study (24).

Laboratory diagnosis of *Aeromonas* infections usually involves the isolation of potential *Aeromonas* species by bacterial cultivation using selective media developed for other enteric pathogens. *Aeromonas* species are then identified using various methods, such as biochemical tests or matrix-assisted laser desorption ionization–time of flight mass spectrometry (MALDI-TOF MS). Currently, limited information is available regarding the use of molecular methods for detecting *Aeromonas* enteric infection in diagnostic laboratories.

In this study, we examined the detection of *Aeromonas* species and four other enteric pathogens by quantitative real-time PCR (qPCR) in 341,330 fecal samples from patients with gastroenteritis in a large Australian diagnostic laboratory. We also compared the cycle threshold (*C_T_*) values obtained from fecal samples that were *Aeromonas* positive only by molecular detection with samples that were positive using both molecular detection and bacterial culture.

## RESULTS

### *Aeromonas* species were the second most common bacterial pathogens in patients with gastroenteritis.

A total of 341,330 stool samples were analyzed between 2015 and 2019 by qPCR. Using commercially available molecular detection methods, the five common enteric bacterial pathogens were identified to the genus level. Campylobacter species were most common, with the detection rate being 512.96 per 10,000 samples. *Aeromonas* species were the second most common, with a detection rate of 255.15 per 10,000 samples. The detection rates for Salmonella, *Yersinia*, and *Shigella*/enteroinvasive Escherichia coli (EIEC) species were 144.52, 57.22, and 50.07 per 10,000 samples, respectively (Table 1).

**TABLE 1:** Quantitative real-time PCR detection of *Aeromonas*, Campylobacter, Salmonella, *Shigella*/EIEC, and *Yersinia* species in fecal samples from patients with gastroenteritis^*a*^

Bacterial species	No. of positive results						
	2015	2016	2017	2018	2019	Total	Detection rate
Campylobacter	2,416	3,451	3,362	3,753	4,527	17,509	512.96
*Aeromonas*	1,381	1,548	1,637	2,045	2,098	8,709	255.15
Salmonella	747	1,153	1,021	1,001	1,011	4,933	144.52
*Yersinia*	318	289	313	505	528	1,953	57.22
*Shigella*/EIEC	237	306	398	377	391	1,709	50.07

a Data were obtained from a total of 341,330 stool specimens tested. EIEC, enteroinvasive E. coli.

### *Aeromonas* enteric infection displayed three infection peaks related to patient age.

**FIG 1:**
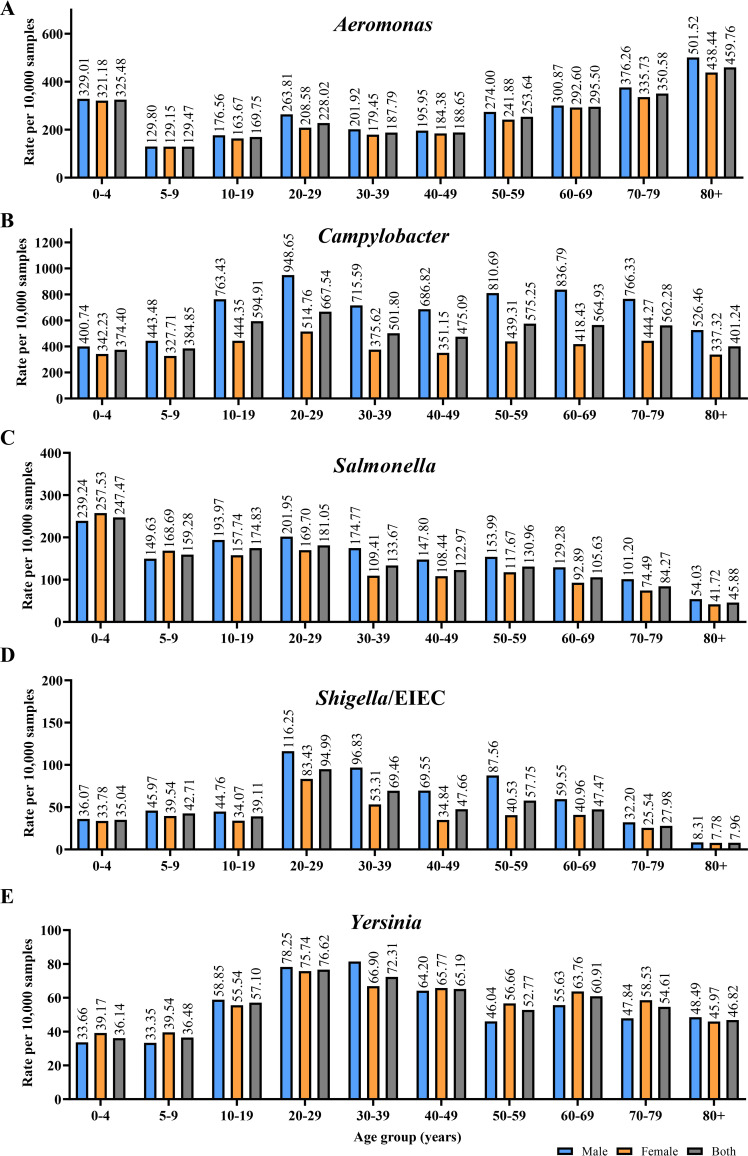
Quantitative real-time PCR detection of *Aeromonas*, Campylobacter, Salmonella, *Shigella*/EIEC, and *Yersinia* species in different age groups, from 0 to 80+ years. The bacterial pathogens were detected in 341,330 fecal samples from patients with gastroenteritis. The detection rates are presented as the number of positive results per 10,000 samples. (A) Three peaks of *Aeromonas* enteric infections were observed, in patients aged 0 to 4 years, 20 to 29 years, and over 50 years old. (B) Positive Campylobacter results peaked in young adults aged 20 to 29 years. (C) Detection rates for Salmonella species peaked in young children aged 0 to 4 years. (D and E) Detection rates for *Shigella*/EIEC (D) and *Yersinia* (E) species were found to peak in young adults aged 20 to 29 years. A positive association between the detection rate and patient age was observed for *Aeromonas*, Campylobacter, and *Yersinia* (*P *< 0.001, *P *< 0.001, and *P* = 0.014, respectively), while a negative association was observed for Salmonella and *Shigella*/EIEC (*P *= 0.987 and *P *< 0.001, respectively). EIEC, enteroinvasive E. coli.

The detection rates of *Aeromonas* species by age groups and gender of the patients are shown in Fig. 1. The *Aeromonas* detection rate in children 0 to 4 years old was high (325.48 per 10,000 samples) and formed the first peak of infection. Detection rates dropped in patients aged 5 to 19 and then increased in those aged 20 to 29 to 228.02 per 10,000 samples, forming the second small peak of infection. The detection rate continuously increased in patients over 50 years old and formed the third infection peak in patients aged over 80 years old (range, 253.64 to 459.76 per 10,000). The association between the *Aeromonas* detection rate and patient age was found to be significant (*P* < 0.001; odds ratio, 1.007; 95% confidence interval, 1.006 to 1.008). Male patients were found to have a higher detection rate than female patients (*P* < 0.001; odds ratio, 1.136; 95% confidence interval, 1.088 to 1.186). The number of positive results and processed samples in each age group are provided in Table 2.

**TABLE 2:** Quantitative real-time PCR detection of *Aeromonas*, Campylobacter, Salmonella, *Shigella*/EIEC, and *Yersinia* species by year (2015 to 2019) and age group

Age (yrs)	No. of positive results for:										No. of samples tested	
	*Aeromonas*		Campylobacter		Salmonella		*Shigella*/EIEC^*a*^		*Yersinia*			
	Male	Female	Male	Female	Male	Female	Male	Female	Male	Female	Male	Female
2015												
0–4	143	100	150	112	78	75	10	9	22	18	3,872	2,973
5–9	30	30	87	55	22	31	12	6	7	6	1,830	1,759
10–19	34	50	122	81	40	32	8	11	12	9	1,609	1,868
20–29	38	107	178	182	49	64	19	33	17	24	1,730	3,350
30–39	58	90	149	162	50	56	18	23	21	43	2,297	4,157
40–49	41	86	150	111	40	31	14	10	8	32	2,071	3,695
50–59	48	95	122	144	23	43	15	12	6	17	1,779	3,157
60–69	48	121	148	149	25	35	11	11	10	21	1,714	3,305
70–79	55	85	101	111	14	21	7	7	9	24	1,346	2,283
80+	39	83	39	63	2	16	0	1	5	7	881	1,838
Total	534	847	1,246	1,170	343	404	114	123	117	201	19,129	28,385
2016												
0–4	147	122	208	151	151	135	18	13	16	17	4,759	4,020
5–9	26	22	115	64	25	35	5	7	12	9	2,129	2,226
10–19	27	35	185	99	45	56	4	12	11	11	2,205	2,482
20–29	71	86	230	242	72	97	37	43	16	24	2,501	4,661
30–39	60	97	247	201	64	80	20	25	20	19	3,202	5,511
40–49	55	83	219	209	58	71	18	18	11	19	2,798	4,846
50–59	73	93	210	222	41	63	21	26	13	20	2,481	4,493
60–69	70	117	184	204	31	44	12	16	13	24	2,335	4,418
70–79	72	107	137	152	21	40	3	5	4	13	1,882	3,275
80+	72	113	71	101	5	19	2	1	3	14	1,331	2,634
2017												
0–4	155	125	181	132	114	106	25	18	17	11	4,998	4,197
5–9	31	28	85	80	38	44	15	11	5	7	2,208	2,271
10–19	34	35	168	108	53	53	12	8	10	13	2,381	2,581
20–29	70	85	236	259	56	93	25	43	16	37	2,717	5,080
30–39	72	104	230	215	57	65	30	45	20	27	3,407	5,780
40–49	50	92	192	184	39	57	23	22	18	28	3,101	5,314
50–59	86	95	198	190	40	49	29	14	8	20	2,725	4,714
60–69	76	128	194	190	31	49	19	23	8	23	2,570	4,799
70–79	79	100	167	174	26	33	12	15	7	16	2,176	3,890
80+	71	121	78	101	10	8	3	6	8	14	1,510	2,938
2018												
0–4	186	158	205	140	130	107	24	14	11	16	5,297	4,269
5–9	26	32	87	82	36	34	8	10	6	9	2,354	2,484
10–19	60	46	204	130	41	40	14	6	18	26	2,804	3,128
20–29	90	133	279	291	47	78	30	40	30	48	3,150	5,725
30–39	74	119	264	213	54	66	49	31	46	58	3,893	6,496
40–49	77	113	219	192	44	57	29	18	29	38	3,349	5,728
50–59	80	123	252	185	54	62	18	23	18	38	3,032	5,176
60–69	87	155	239	198	39	42	16	27	16	31	2,925	5,301
70–79	105	150	178	201	22	26	6	11	20	27	2,631	4,357
80+	84	147	89	105	12	10	1	2	9	11	1,645	3,156
2019												
0–4	190	151	256	164	124	103	13	15	18	18	6,028	4,966
5–9	31	35	118	92	45	48	11	11	7	14	2,573	2,642
10–19	58	55	242	182	55	32	16	9	20	16	3,065	3,444
20–29	85	104	350	297	47	87	45	47	26	54	3,321	5,875
30–39	78	105	322	287	71	47	47	29	31	45	4,138	6,755
40–49	70	97	247	201	40	61	20	21	30	51	3,634	5,962
50–59	76	149	292	267	46	53	33	18	16	35	3,231	5,405
60–69	103	172	303	250	39	50	18	20	24	52	3,219	5,861
70–79	98	189	250	197	27	20	7	10	12	30	2,835	4,990
80+	96	156	103	107	10	6	0	1	10	19	1,851	3,575

a EIEC, enteroinvasive E. coli.

The Campylobacter detection rate displayed a peak at 667.54 per 10,000 samples in young adults aged 20 to 29 years. There was an association between the Campylobacter detection rate and patient age (*P* < 0.001; odds ratio, 1.003; 95% confidence interval, 1.002 to 1.004). The detection rate in male patients was found to be significantly higher (*P* < 0.001; odds ratio, 1.752; 95% confidence interval, 1.699 to 1.807).

The detection rate for Salmonella species was found to have a peak in young children 0 to 4 years old (247.47 per 10,000 samples). The association between the Salmonella detection rate and patient age was found to be significant (*P* < 0.001; odds ratio, 0.987; 95% confidence interval, 0.986 to 0.988). Male patients were found to have a significantly higher detection rate than female patients (*P* < 0.001; odds ratio, 1.194; 95% confidence interval, 1.128 to 1.264).

For *Shigella*/EIEC and *Yersinia* species, the detection rates were observed to peak in young adults aged 20 to 29 years old. The *Shigella*/EIEC detection rate was found to be associated with patient age (*P* < 0.001; odds ratio, 0.996; 95% confidence interval, 0.994 to 0.998). The *Shigella*/EIEC detection rate was also found to be higher in male patients (*P* < 0.001; odds ratio, 1.439; 95% confidence interval, 1.307 to 1.584). The *Yersinia* detection rate was associated with patient age (*P* = 0.014; odds ratio, 1.002; 95% confidence interval, 1 to 1.004). However, patient gender was not found to be associated with the *Yersinia* detection rate.

### *Aeromonas* species were the most common enteric bacterial pathogen in children younger than 18 months.

**FIG 2:**
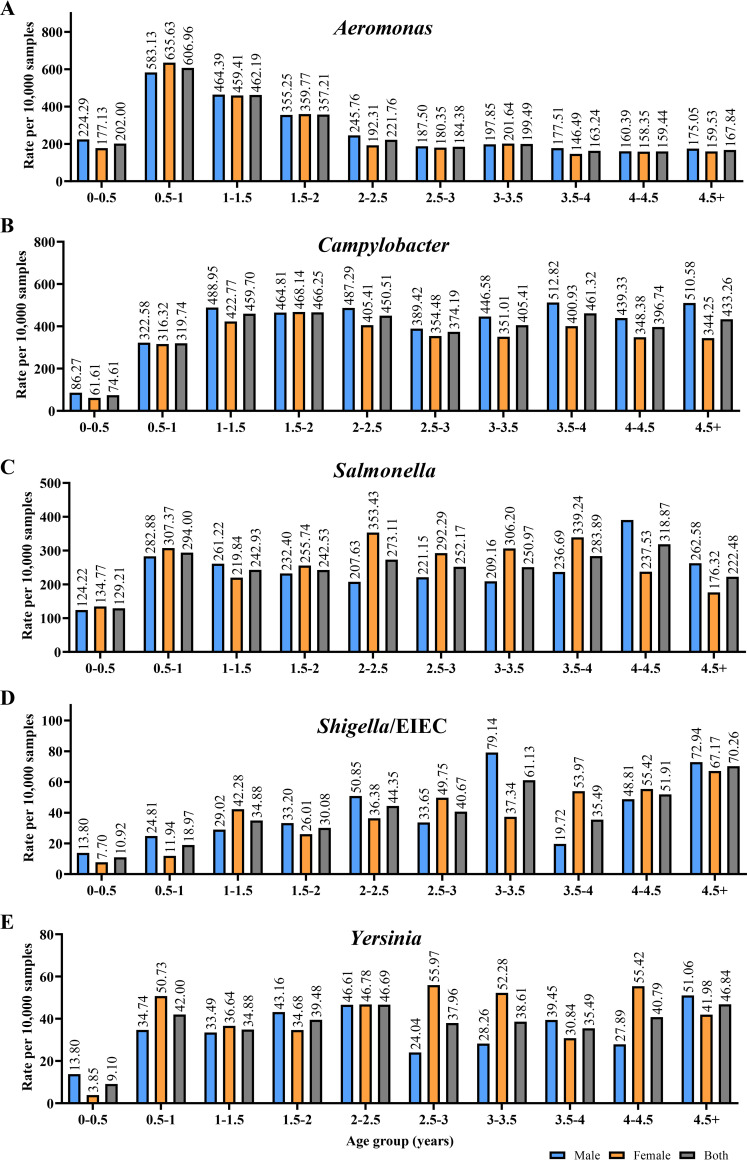
Quantitative real-time PCR detection of *Aeromonas*, Campylobacter, Salmonella, *Shigella*/EIEC, and *Yersinia* species in children with gastroenteritis aged 0 to 4 years. (A) *Aeromonas* enteric infection was common in children 6 to 24 months old. In children younger than 18 months old, *Aeromonas* species were the most common enteric pathogens. (B to E) Rates of infection with Campylobacter (B), Salmonella (C), *Shigella*/EIEC (D), and *Yersinia* (E) enteric pathogens in children 0 to 4 years old. EIEC, enteroinvasive E. coli.

Molecular detection revealed an *Aeromonas* enteric infection peak in children 0 to 4 years old. This infection peak was not seen in our previous report of *Aeromonas* enteric infection detection by bacterial culture in the Douglass Hanly Moir (DHM) laboratory during the same years. We therefore conducted further analysis by grouping these children based on 6-month age intervals. Interestingly, *Aeromonas* species showed a novel infection pattern compared to those of other enteric pathogens. *Aeromonas* enteric infections were common in children aged 6 to 24 months old. In children younger than 18 months old, *Aeromonas* species were the most common enteric bacterial pathogens (Fig. 2). The detection rates of *Aeromonas* species and four other enteric pathogens in male and female children less than 2 years old were not statistically different (*P *> 0.05).

### Fecal samples positive for *Aeromonas* only by molecular detection had significantly higher mean *C_T_* values than fecal samples positive by both molecular detection and *Aeromonas* culture.

The mean *C_T_* value of the 235 fecal samples that were positive using qPCR detection but negative using *Aeromonas* culture was 32.5, which was significantly higher than the mean *C_T_* value (29.03) of the 45 fecal samples that were positive by both qPCR detection and *Aeromonas* culture (*P* < 0.001; 95% confidence interval, 1.9 to 5.04).

### Examination of false-negative qPCR results among samples with positive *Aeromonas* and other enteric bacterial culture.

The false-negative qPCR results among samples with positive bacterial enteric pathogens varied among different bacterial species. *Aeromonas* had the highest false-negative rate (11.73%), followed by Salmonella (8.75%). The false-negative rates for Campylobacter and *Shigella* were low, at 1.1% and 0.1%, respectively (Table 3).

**TABLE 3:** False-negative qPCR results among samples with a positive bacterial culture

Genus	No. of positive samples by bacterial culture^*a*^	No. of negative samples by qPCR	False-negative rate (%)
*Aeromonas*	2,132	250	11.73
Campylobacter	11,597	127	1.1
Salmonella	5,199	455	8.75
*Shigella*	257	2	0.1
*Yersinia*	71	6	8.45

a The number of positive samples by bacterial culture were reported previously (25).

## DISCUSSION

*Aeromonas* species are increasingly being recognized as human enteric pathogens. In this retrospective study, we investigated the presence of *Aeromonas* species and four other enteric bacterial pathogens detected using molecular detection methods. Our analysis involved a large number of fecal samples from patients with gastroenteritis. The findings from this study provide novel and valuable information regarding laboratory diagnosis of *Aeromonas* enteric infections.

*Aeromonas* species were the second most identified bacterial pathogens by molecular detection in patients with gastroenteritis. The most common enteric bacterial pathogen species detected were Campylobacter species (Table 1). Notably, molecular detection of *Aeromonas* enteric infection revealed a distinctive pattern characterized by three infection peaks, a unique feature not observed in the other four bacterial enteric pathogens tested (Fig. 1). The occurrence of *Aeromonas* enteric infections was predominantly observed in young children and individuals over 50 years old (Fig. 1), suggesting a higher susceptibility to these infections during stages of relative immune weakness. Additionally, there was an increase in *Aeromonas* enteric infections among patients aged 20 to 29 years, possibly attributed to increased exposure to *Aeromonas* pathogens. Our previous research demonstrated that the majority (96%) of *Aeromonas* enteric infections in Australia were locally acquired, with no history of overseas travel (25). Future investigations are needed to identify the sources of *Aeromonas* enteric infections in Australia; effective strategies can then be implemented to reduce these infections.

Further analysis of enteric infections in children aged 0 to 4 years revealed that *Aeromonas* species were the most common bacterial enteric pathogens in children younger than 18 months old (Fig. 2). Previous studies have also reported cases of *Aeromonas*-associated gastroenteritis in children. For example, a prospective study conducted by Gracey et al. in 1982 in Western Australia examined the isolation of *Aeromonas* species from fecal samples of 1,156 children with gastroenteritis, with an equal number of age- and sex-matched controls. The study found *Aeromonas* species in 10.2% of children with diarrhea and 0.6% of controls (26). The most common presentation was self-limiting diarrhea, although 25% of patients exhibited dysentery-like symptoms (26). Consistent with our findings, Gracey et al. also found that *Aeromonas* enteric infections were most prevalent in children less than 2 years old (26). Another study by Qamar et al. in 2016 reported that *Aeromonas* was a significant pathogen (22.2%) for moderate to severe diarrhea in children under 5 years old in Pakistan and Bangladesh (27). Multiple studies from various global regions have also reported *Aeromonas*-associated gastroenteritis in children (28–33). The findings from our current study, along with previous research, consistently support the importance of *Aeromonas* species as important enteric pathogens in young children.

The molecular detection methods used in this study generally exhibited higher sensitivity in detecting enteric pathogens compared to bacterial isolation methods. However, the degree of improvement varied for different pathogens. The detection rate of *Aeromonas* species using the qPCR method in this study was 255.15 per 10,000 samples, representing a 4.5-fold increase compared to the detection rate by *Aeromonas* isolation (56.73/10,000 samples) from fecal samples of patients with gastroenteritis at DHM during the same period (25). Similarly, the molecular detection rates for Campylobacter, Salmonella, and *Yersinia* in this study were 512.96, 144.52, and 57.22 per 10,000 samples, respectively, representing 1.66-, 1.04-, and 30.26-fold increases compared to bacterial isolation, respectively (25). It is worth noting that not all samples that tested positive by bacterial culture yielded positive results by qPCR. The false-negative rates of qPCR among samples with positive bacterial cultures varied from 0.1 to 11.73%. These findings show the importance of utilizing both bacterial culture and qPCR to enhance the detection of enteric pathogens.

Our analysis shows that fecal samples that were positive only by molecular detection of *Aeromonas* species had a significantly higher *C_T_* value from qPCR than fecal samples that were positive by both molecular detection and bacterial culture. This observation indicates that samples with lower quantities of *Aeromonas* bacteria were more likely to yield negative results in *Aeromonas* culture. Currently, the isolation of *Aeromonas* species from fecal samples in diagnostic laboratories primarily relies on selective media designed for other enteric bacterial pathogens (25). Our results suggest that *Aeromonas*-specific selective media should be developed to enhance the *Aeromonas* isolation rate in diagnostic laboratories.

In summary, we report that *Aeromonas* species were the second most common bacterial enteric pathogen species in patients with gastroenteritis in an Australian population, as detected by molecular methods. Our study also reveals three distinct peaks of *Aeromonas* enteric infection that are novel and age related. Furthermore, we show that *Aeromonas* species were the most common enteric bacterial pathogen species in children younger than 18 months. Currently, *Aeromonas* enteric pathogens are not routinely tested in diagnostic laboratories. The high rate of *Aeromonas* enteric infection discovered in our study and the importance of *Aeromonas* affecting different patient age groups suggest that *Aeromonas* species should be included on the common enteric bacterial pathogen examination list in diagnostic laboratories. Our data also show that utilizing both bacterial culture and qPCR can improve the detection of enteric pathogens.

## MATERIALS AND METHODS

### Clinical data of enteric bacterial pathogens detected using molecular methods.

The data analyzed in this study were provided by the Douglass Hanly Moir (DHM) pathology laboratory in Sydney, Australia. From 2015 to 2019, a total of 341,330 stool samples from patients with gastroenteritis were tested for bacterial enteric pathogens using qPCR methods, including *Aeromonas*, Campylobacter, Salmonella, *Shigella* EIEC, and *Yersinia*. Fecal samples mixed in stool transport and recovery (STAR) buffer were extracted using a MagnaPure 96 (Roche, Basel, Switzerland) instrument, and qPCR was performed on the LightCycler 480 II (Roche) system using the LightMix gastro bacteria kit manufactured by TIB Molbiol (Berlin, Germany) for the detection of *Aeromonas*, Campylobacter, Salmonella, *Shigella*/EIEC, and Yersinia enterocolitica. *C_T_* cutoff values of ≤37 (≤38 for *Aeromonas*) were considered positive if associated with an amplification curve. At DHM, one fecal sample from each case was used for molecular detection of enteric pathogens.

### Examination of the detection rates of *Aeromonas*, Campylobacter, Salmonella, *Shigella*/EIEC, and *Yersinia* species in patients with gastroenteritis.

The data of detection of the five enteric bacterial pathogens using qPCR methods at the DHM laboratory over 5 years (2015 to 2019) were analyzed. The detection rate for each enteric bacterial pathogen (positive results per 10,000 samples) was calculated based on the 5-year data.

### Comparison of the detection rates for *Aeromonas*, Campylobacter, Salmonella, *Shigella*/EIEC, and *Yersinia* species in patients of different age groups.

The patients were grouped into different age groups. The number of processed fecal samples in each of the age groups and the number of positive results were recorded. Detection rates were calculated for the five enteric bacterial pathogens in each of the age groups. Logistic regression analyses were performed using a binomial generalized linear model to assess the relationship between the isolation rates of enteric pathogens and patients’ age or gender. The statistical analyses were performed by using R v. 4.0.4 software and RStudio v. 1.4.1106. A *P* value of <0.05 was considered statistically significant.

### Comparison of qPCR *C_T_* values.

At the DHM, fecal samples were tested using both molecular detection and bacterial isolation of *Aeromonas.* To examine whether *Aeromonas* bacterial numbers in fecal samples are a potential factor contributing to *Aeromonas* isolation positivity, we compared the *C_T_* values of *Aeromonas* qPCR detection from fecal samples that were only positive by molecular detection with those of fecal samples that were positive by both molecular detection and *Aeromonas* culture. The qPCR *C_T_* values were available for 280 *Aeromonas*-positive fecal samples detected in the year 2021. Of the 280 fecal samples, 45 samples were positive by both qPCR detection and bacterial cultivation; the remaining 235 fecal samples were positive only by qPCR detection but negative for *Aeromonas* isolation. The mean *C_T_* values of the samples of these two groups were compared using Welch’s *t* test.

For *Aeromonas* cultivation from fecal samples, the presumptive *Aeromonas* isolates were gathered from three different types of culture plates, which included xylose lysine deoxycholate (XLD) agar, thiosulfate-citrate-bile salts-sucrose (TCBS) agar, and horse blood agar (HBA) containing an ampicillin AMO25 disc (Oxoid, Scoresby, VIC, Australia), based on the appearance of the colonies, as described previously (25). The isolates were then subjected to an oxidase test. Those that tested positive for oxidase were subsequently identified using matrix-assisted laser desorption ionization–time of flight mass spectrometry (MALDI-TOF MS). The results were reported as “*Aeromonas* species,” without specifying the species due to the possible error in identifying *Aeromonas* beyond the genus level by MALDI-TOF MS.

### Examination of false-negative qPCR results among samples with positive *Aeromonas* and other enteric bacterial culture.

Previously, we reported the detection of *Aeromonas* and other enteric bacterial pathogens by bacterial culture at the DHM between 2015 and 2019 (25). These culture-positive samples were also screened for enteric pathogens by qPCR methods. Here, we examined the occurrence of false-negative qPCR results among samples that tested positive by bacterial culture.

### Ethics approval.

The use of bacterial detection data provided by DHM for this retrospective study, which did not involve patient consent, was approved by the University of New South Wales HREAP executive (HC200755).
